# Estimation of secondary cancer projected risk after partial breast irradiation at the 1.5 T MR-linac

**DOI:** 10.1007/s00066-022-01930-5

**Published:** 2022-04-12

**Authors:** C. De-Colle, O. Dohm, D. Mönnich, M. Nachbar, N. Weidner, V. Heinrich, S. Boeke, C. Gani, D. Zips, D. Thorwarth

**Affiliations:** 1 Department of Radiation Oncology, University Hospital and Medical Faculty, Eberhard Karls University Tübingen, Hoppe-Seyler-Str. 3, 72076 Tübingen, Germany; 2 Section for Biomedical Physics, Department of Radiation Oncology, University Hospital and Medical Faculty, Eberhard Karls University Tübingen, Tübingen, Germany; 3 partner site Tübingen, and German Cancer Research Center (DKFZ), German Cancer Consortium (DKTK), Heidelberg, Germany

**Keywords:** Breast cancer, Partial breast irradiation, Magnetic resonance guided linear accelerator, Second cancer risk, Radiation-induced secondary malignancies

## Abstract

**Purpose:**

For patients treated with partial breast irradiation (PBI), potential long-term treatment-related toxicities are important. The 1.5 T magnetic resonance guided linear accelerator (MRL) offers excellent tumor bed visualization and a daily treatment plan adaption possibility, but MRL-specific electron stream and return effects may cause increased dose deposition at air–tissue interfaces. In this study, we aimed to investigate the projected risk of radiation-induced secondary malignancies (RISM) in patients treated with PBI at the 1.5 T MRL.

**Methods:**

Projected excess absolute risk values (EARs) for the contralateral breast, lungs, thyroid and esophagus were estimated for 11 patients treated with PBI at the MRL and compared to 11 patients treated with PBI and 11 patients treated with whole breast irradiation (WBI) at the conventional linac (CTL). All patients received 40.05 Gy in 15 fractions. For patients treated at the CTL, additional dose due to daily cone beam computed tomography (CBCT) was simulated. The t‑test with Bonferroni correction was used for comparison.

**Results:**

The highest projected risk for a radiation-induced secondary cancer was found for the ipsilateral lung, without significant differences between the groups. A lower contralateral breast EAR was found for MRL-PBI (EAR = 0.89) compared to CTL-PBI (EAR = 1.41, *p* = 0.01), whereas a lower thyroid EAR for CTL-PBI (EAR = 0.17) compared to MRL-PBI (EAR = 0.33, *p* = 0.03) and CTL-WBI (EAR = 0.46, *p* = 0.002) was observed. Nevertheless, when adding the CBCT dose no difference between thyroid EAR for CTL-PBI compared to MRL-PBI was detected.

**Conclusion:**

Better breast tissue visualization and the possibility for daily plan adaption make PBI at the 1.5 T MRL particularly attractive. Our simulations suggest that this treatment can be performed without additional projected risk of RISM.

**Supplementary Information:**

The online version of this article (10.1007/s00066-022-01930-5) contains supplementary material, which is available to authorized users.

## Introduction

Breast cancer is the most common tumor worldwide, representing 11.7% of total cancer diagnoses with an average world incidence among women of almost 50 per 100,000, which increases to 90 for northern and western Europe [1]. More than three-quarters of cases are diagnosed in an early stage, with 5‑year overall survival rates higher than 90% and an increasing curability trend [2]. In this patient group, a major goal of clinical research is to reduce treatment-related toxicities without compromising oncological outcomes. Radiotherapy is a standard treatment after breast-conserving surgery. Because of the high number of long-term breast cancer survivors, radiation-induced secondary malignancies (RISM) represent a major concern. As part of treatment de-escalation strategies, during the last few years partial breast irradiation (PBI) has become a recommended treatment option for selected early stage low-risk breast cancer patients [3–9].

Recently, linear accelerators (linac) with an integrated magnetic resonance (MR) image-guidance device have been introduced into clinical practice [10–17]. Compared to conventional radiotherapy techniques, MR offers better visualization of the breast tissue, the tumor bed and the postoperative changes [18]. In addition, MR-linear accelerators (MRL) allow a MR-guided daily plan adaption. In 2019, the first breast cancer patients were successfully treated at the 1.5 T MRL [16, 17]. Besides advantages, challenging aspects of this new technology must be considered. Due to the Lorentz force, MRL-specific electron stream effect (ESE) and electron return effect (ERE) may cause an increased in-field and also out-of-field dose deposition, especially at air–tissue interfaces. Because of the target location, for breast cancer patients these effects have been described on the chin and the ipsilateral arm of the patient for the ESE as well as on the interface lung/thoracic wall and the skin in the target region which were related to ERE [16, 17, 19–23]. The goal of the present work was to investigate the projected risk of RISM in patients treated with PBI at the 1.5 T MRL and compare it with breast cancer patients treated at the conventional linac (CTL).

## Materials and methods

### Patient selection and treatment modalities

The present study was approved by the ethics committee of the Medical Faculty, University of Tübingen (number 585/2021BO2). The projected risk of RISM was estimated for 11 patients treated with PBI at the 1.5 T MRL Unity (Elekta AB; Stockholm, Sweden), for 11 patients treated with PBI at the CTL (Synergy, Elekta AB, Stockholm, Sweden) and for 11 patients treated with whole breast irradiation (WBI) at the CTL. Patients were selected for PBI based on the Groupe Europeen de Curietherapie – European Society for Therapeutic Radiology and Oncology (GEC-ESTRO) [7] and the updated American Society for Radiation Oncology (ASTRO) criteria [8], namely older than 50 years, with unicentric, unifocal invasive hormone-positive, Her2-negative, grade 1–2 not lobular breast cancer without extensive intraductal component, without lymphovascular invasion, with proliferation index < 25% and resected with a minimum of 2 mm. In August 2018, we implemented in our department PBI treatments as standard therapy for patients meeting the above-mentioned criteria. In September 2018, we started our clinical activity at the MRL. From January 2019 until April 2019, patients who met the PBI criteria were treated at the MRL. In this period, we treated 11 patients with PBI at the MRL. For comparison, we selected the first 11 patients treated with PBI at the CTL, namely from August 2018 until January 2020. During the period January–April 2019, 4 patients suitable for PBI were treated at the CTL and not at the MRL for the following reasons: 1 patient because of claustrophobia, 1 patient refused to enter the MRL study and 2 patients because of obesity. In January 2020, we implemented treatments using the intensity-modulated radiation therapy (IMRT) technique for breast cancer patients receiving WBI. For comparison with PBI MRL and CTL, we selected the first 11 patients treated with WBI IMRT. Excluded were patients with boost to the tumor bed and/or receiving irradiation of both breasts. Since 2 patients were treated with PBI at the CTL and not at the MRL because of obesity, the body mass index (BMI) of patients in the three groups was calculated to assess possible selection bias.

The planning computed tomography (CT) was performed with 3 mm thick slices from skull base to diaphragm. The Treatment Planning System Monaco 5.4 (Elekta AB, Stockholm, Sweden) was used for IMRT planning for PBI at the MRL, PBI at the CTL and WBI. MRL planning and treatment details have been previously described [16, 17]. Briefly, the clinical target volume (CTV) included clips, seroma and visible postoperative changes. Since all patients included in our study received oncoplastic surgery, the CTV for PBI defined as described above resulted in a large volume. Therefore, no additional margins for a CTV2 were added. The planning target volume (PTV) was obtained by 10 mm CTV expansion, limited to 5 mm towards the skin and 7 mm posteriorly, according to our institutional standards. Patients were treated with 40.05 Gy in 15 fractions using a step-and-shoot IMRT technique with 5 to 7 beams with a daily MR-based plan adaption and an intrafractional MR-based motion monitoring during “beam on” time. CTV and PTV for PBI at the CTL were defined as for PBI at the MRL. The CTV for WBI was defined according to ESTRO delineation guidelines [24], namely borders were considered: cranial below the medial head of clavicle, caudal the most inferior slice with breast tissue visible, lateral the thoracic artery, medial the mammarian vessels, anterior 5 mm under the skin and posterior the anterior surface of pectoralis major. The PTV was created as for PBI. Patients treated with WBI received 40.05 Gy in 15 fractions, as the PBI patients. Plans for treatments at the CTL consisted of volumetric modulated arc intensity-modulated radiotherapy (VMAT-IMRT, 0–180° and 180–0° according to right or left involved breast side) for PBI and dynamic multileaf collimator (dMLC-IMRT) with four beams for WBI. All treatments were performed with isocentric beams. For PBI at the CTL, image-guidance consisted of a daily cone-beam CT (CBCT). Details on the CBCT imaging protocol can be found in supplementary table 1. Examples of dose distributions for PBI treatment at the MRL and CTL are shown in supplementary figure 1.

### Risk estimation

For the projected risk estimation of RISM organ equivalent dose (OED) model was used [25]. Briefly, OED is defined as the equivalent uniformly distributed dose in a specific organ which is associated with the same radiation-induced cancer incidence. In this model, two components of the dose–response relationship for RISM are considered, namely the low- and the high-dose component. For low doses, the dose–response relationship for carcinogenesis is assumed to be linear, while at higher doses (as those applied in radiotherapy) the component of cell killing becomes relevant. In addition, in radiotherapy usually the dose received by an organ is not uniform. For example, for breast cancer patients, the part of the ipsilateral lung located close to the target receives the highest dose (with tangential techniques up to 100% of the prescribed dose), while the rest of the lung receives only very limited dose. Because of the two components of the dose–response relationship for carcinogenesis, it would not be correct to use the organ average dose for the risk estimation. For each organ, the OED can be estimated based on the dose–volume histogram (DVH) using the organ-specific cell sterilization parameter α. The α values for lung (0.129), thyroid (0.033) and esophagus (0.274) used in this evaluation were taken from the literature [25], while for breast (0.25) the α value was estimated according to Schneider et al. [26]. The excess absolute risk (EAR) represents the absolute difference in cancer rates of persons exposed to radiation and those not exposed. Knowing the OED and the organ-specific risk coefficient µ, it is possible to estimate the projected EAR. The risk coefficients were taken from the literature [27] and were 9.47 for lung, 0.86 for thyroid, 1.9 for esophagus and 2.49 for breast. The procedure used is described in detail by other authors [28]. EARs are expressed per 10,000 persons/year. For example, an EAR value of 1 means that one in 1000 treated patients will develop an additional RISM during a period of 10 years. In our study, EARs for the contralateral breast, lungs (both ipsilateral and contralateral), thyroid and esophagus were estimated. After testing for normal distribution with Shapiro’s test, t‑test with Bonferroni correction was used for EARs comparison between the groups. A comparison between the PTVs and the dose to the contralateral breast was performed using the t‑test.

In addition, patients treated with PBI at the CTL received a daily CBCT, which might also contribute to increase the risk of RISM. We therefore simulated the dose deposited in patients by a daily CBCT using an in-house developed method based on Monte Carlo calculations. Briefly, a BEAMnrc [29] model of the CBCT, calibrated to absolute dose for the respective CBCT protocol, was applied on the patient’s planning CT. The resulting dose distribution was then summed with the patient’s treatment plan for total dose distribution. We incorporated these values into our model for risk estimation generating cumulative DVHs considering 15 CBCTs. Patients treated with WBI at the CTL did not receive a homogeneous number of CBCT, following our institutional standard of a daily CBCT during the first 3 fractions and subsequently a patient-adapted CBCT frequency according to individual positioning reproducibility. Therefore, we did not consider the additional CBCT dose (highly variable from patient to patient, according to CBCTs number) for the projected risk of RISM for WBI patients. Nonetheless, we reported the impact of exemplarily 6 CBCT (according to our clinical practice, where patients receive at least 3 CBCTs the first week, 2 the second week, 1 the last week) on the mean dose of the different organs at risk (OARs).

## Results

**Fig. 1:**
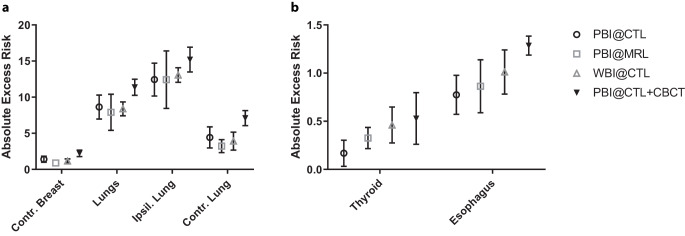
Comparison between excess absolute risk for **a** contralateral breast and lungs (both ipsilateral and contralateral), **b** thyroid and esophagus. EAR values for thyroid and esophagus lay in the very low dose range, scale on the x‑axis is therefore different between **a** and **b**

The highest projected risk for developing a radiation-induced second cancer was found for the ipsilateral lung, with mean EARs of 12.4, 12.4 and 13.1 for PBI at the MRL, PBI at the CTL and WBI at the CTL, respectively (Fig. 1a, Table 1). A lower contralateral breast EAR was found for PBI at the MRL compared with PBI at the CTL (*p* = 0.01, Fig. 1a, Table 1). Without considering the dose due to the CBCTs, a lower thyroid EAR for PBI at the CTL (EAR = 0.17) compared to PBI at the MRL (EAR = 0.33, *p* = 0.03) and WBI at the CTL (EAR = 0.46, *p* = 0.002, Fig. 1b, Table 1) was found in the very low dose range. No difference was observed for all other organs. When considering the additional dose due to the CBCTs for PBI treatments at the CTL, no difference could be detected between thyroid EAR values for PBI at the CTL and at the MRL. A significant difference between EAR values for PBI at the CTL and at the MRL in favor of MRL was detected for contralateral breast (*p* < 0.0001), both lungs (*p* = 0.004), contralateral lung (*p* < 0.0001) and esophagus (*p* = 0.002, Table 1). By adding the dose of the daily CBCTs, there was an increase of the mean dose of about half a Gray on all organs considered for PBI patients treated at the CTL (contralateral breast: 0.55 Gy, both lungs: 0.52; ipsilateral lung: 0.66 Gy, contralateral lung: 0.36 Gy; thyroid: 0.51 Gy, esophagus: 0.45 Gy, table 3 supplementary material). For WBI patients, by adding the dose of a total of 6 CBCTs there was an increase of the mean dose of about 0.2 Gy for all organs considered (contralateral breast: 0.22 Gy, both lungs: 0.23 Gy; ipsilateral lung: 0.26 Gy, contralateral lung: 0.15 Gy; thyroid: 0.15 Gy, esophagus: 0.2 Gy, cf. supplementary table 4). To further investigate the difference in EAR values for the contralateral breast between PBI at the MRL and at the CTL, which was not observed between PBI at the MRL and WBI at the CTL, we performed a comparison of the PTVs between PBI at the MRL and at the CTL, which did not show any significant difference (mean, range: 265.3 cm^3^, 83.5–539.6 cm^3^ with MRL vs*.* 299 cm^3^, 82.2–615 cm^3^ with CTL, *p* = 0.6, table 2 and figure 2 of the supplementary material) and a comparison between the maximum dose to the contralateral breast, which revealed higher values for the WBI compared to PBIs (mean, range: 2.86 Gy, 1–10.4 Gy for PBI at the MRL, 3.7 Gy, 1.9–7.7 Gy for PBI at the CTL and 9.06 Gy, 2.6–19.8 Gy for WBI, table 2 and Fig. 3 of the supplementary material). There were no differences in the BMI between the three groups, with mean (range) BMI of 26.2 (20–37.1) for PBI at the MRL vs*.* 26.8 (21.6–36.5) for PBI at the CTL vs. 26.5 (20.3–45.2) for WBI at the CTL.

**Table 1:** Comparison between excess absolute risk values

	PBI@CTL	PBI@MRL	WBI@CTL	PBI@CTL + CBCT	PBI@CTL vs PBI@MRL	PBI@CTL vs WBI@CTL	WBI@CTL vs PBI@MRL	PBI@CTL + CBCT vs PBI@MRL
	M ± SD	M ± SD	M ± SD	M ± SD	*p*-value^a^	*p*-value^a^	*p*-value	*p*-value^a^
Breast contr.	1.408 ± 0.413	0.894 ± 0.241	1.16 ± 0.306	2.222 ± 0.454	**0.01**	0.507	0.142	**<** **0.0001**
Lungs	8.6351 ± 1.656	7.912 ± 2.497	8.391 ± 0.958	11.372 ± 1.112	1.737	2.714	2.249	**0.004**
Lung ipsil.	12.441 ± 2.276	12.423 ± 3.987	13.064 ± 1.01	15.207 ± 1.715	3.96	1.681	2.46	0.213
Lung contr.	4.432 ± 1.463	3.226 ± 0.907	3.92 ± 1.241	7.104 ± 1.042	0.132	1.547	0.606	**<** **0.0001**
Thyroid	0.168 ± 0.136	0.326 ± 0.11	0.461 ± 0.187	0.529 ± 0.268	**0.029**	**0.002**	0.219	0.177
Esophagus	0.774 ± 0.203	0.863 ± 0.275	1.011 ± 0.229	1.286 ± 0.099	1.605	0.074	0.736	**0.002**

*M* mean, *SD* standard deviation, *@* at the

^a^Values in bold are statistically significant differences

## Discussion

The number of patients treated with MRLs is rapidly increasing because of the advantages represented by a better soft tissue visualization and the possibility of a daily MR-based plan adaption. These advantages might also be exploited for breast cancer patients, particularly in the PBI setting. First treatments of adjuvant PBI at the 1.5 T MRL have been successfully performed [16, 17] and numerous protocols worldwide are currently open for breast irradiation at the MRL in the adjuvant as well as neoadjuvant setting [30–36]. Besides clear advantages, MRL-specific challenges, such as the ESE and ERE, must be considered. We have already demonstrated that with the use of a 1 cm bolus placed on the patient’s chin and, when required, on the ipsilateral arm, the MRL-specific out-of-field dose due to the ESE can be effectively avoided [17]. Regarding the ERE, we could observe that the maximum skin dose is increased by less than 1% of the prescribed dose [16], suggesting that treatments at the 1.5 T MRL are safe and associated with, as expected, very low acute and early late toxicities [17]. In the present study, we estimated the projected risk of RISM for patients treated with PBI at the MRL for the contralateral breast, lungs, esophagus and thyroid and compared these results with breast treatments performed at the CTL. In order to have comparable treatments groups, we used the same margin for the PTV for PBI at the MRL and at the CTL. Nevertheless, MR planning and daily MR-based plan adaption possibilities allow for margin reductions, which might reduce the dose to the OARs. We did not observe any difference in the EARs for the lungs (both ipsilateral and contralateral) nor for the esophagus between the three groups of PBI at the MRL, PBI at the CTL and WBI at the CTL. Regarding the contralateral breast, we obtained lower EAR values for PBI at the MRL compared to CTL. We investigated whether these results might have been influenced by differences in target volumes. In fact, through better tumor bed visualization in the MR images, we might have delineated smaller target volumes when performing treatments at the MRL, even though we used the same protocols for the definition of CTV and PTV for PBI performed at the MRL and CTL. The mean target volume was slightly smaller for PBI at the MRL, though the difference was not significant. A difference in contralateral breast EAR values was not seen between PBI at the MRL and WBI at the CTL. Here, it should be considered that the maximal dose in the contralateral breast was significantly higher for WBI and this might influence the calculation of the OED, according to the two components of dose–response relationship for RISM. We observed higher EAR values for the thyroid for PBI at the MRL compared to PBI at the CTL. Here, a component due to the ESE for treatments at the MRL cannot be excluded. Nevertheless, these values are in the very low dose range and lower than those for WBI at the CTL. Moreover, no difference in thyroid EAR values remains detectable when the dose contribution of daily CBCTs is considered for PBI patients treated at the CTL.

Data report an additional dose of up to 2% of the prescribed dose due to daily IGRT for treatments with total dose of 70–80 Gy. Monaco-based calculations showed that a single CBCT for breast cancer treatment deposits an additional dose of up to 5 cGy in the target area and up to 3 cGy to the lung and the rest of the body [37]. In our patient cohort, those treated with PBI at the CTL received a daily CBCT. Considering the additional dose of 15 CBCTs, EAR values were significantly higher than without CBCT. For the considered organs, daily CBCT increased the mean dose by approximately 0.5 Gy (1.2% of the prescribed dose). When the dose of the CBCT is added to the radiotherapy plan dose, no difference in the EAR for the thyroid between PBI at the MRL and PBI at the CTL was detected, whereas significantly lower EAR values were observed for PBI performed at the MRL for all other organs except the ipsilateral lung. We performed PBI according to the IMPORT LOW protocol [6]. Nevertheless, in the last few years, evidence has been provided for external beam PBI in 5 fractions [38] and even though the UK FAST and FAST forward trials [39, 40] investigated 5 fractions for WBI, the FAST forward schedule has started to be used as standard PBI schedule in some countries. PBI in 5 fractions might offer, along with logistical advantages, a reduction in the additional dose due to the CBCT, sparing ten fractions compared to the IMPRT LOW protocol.

All breast cancer patients, independent of radiotherapy, have a higher risk to develop second cancers, most commonly of the lung, esophagus and thyroid compared to the general population [41, 42]. This risk increases considerably for irradiated patients [43]. Surveillance, epidemiology, and end results (SEER) data analysis indicated for breast cancer patients an absolute excess risk for all types of second cancers of 35 per 10,000 patient–years for irradiated patients compared to 23 for non-irradiated patients, for approximately 3.4% of all types of secondary malignancies and 0.8% of contralateral breast cancers attributable to RT [44]. The risk of RISM after breast irradiation has been investigated in plan comparison studies and in phantoms. Our results appear in accordance with the literature [28], where the highest EAR values for the ipsilateral lung have also been reported [45]. Nevertheless, EAR values appear to vary considerably according to the techniques used [46]. In a phantom study, accelerated PBI appeared to reduce the risk of secondary cancer 2‑ to 4‑fold compared to WBI [47], even though these findings were not confirmed in another combined analysis of clinical data from different studies [48]. Generally, EAR calculations are subject to high uncertainties. Not only the dose calculation, but also the cell sterilization parameters and the risk coefficients often contain large uncertainties. Therefore, the EAR values should be considered as an estimation of a projected excessive risk, rather than precise absolute values. Nonetheless, within one organ the excessive risk between different patient groups (PBI at the MRL, PBI at the CTL, WBI at the CTL) is comparable, as for the specific organ considered the same cell sterilization parameter and risk coefficient are used. For dose calculations, Monte Carlo systems were used, i.e., a commercial planning system (Elekta Monaco) to calculate the dose distribution of the treatment plan and a general purpose Monte Carlo system (BEAMnrc) to calculate the CBCT dose distribution. With both systems, the statistical uncertainty inside the field was kept below 1%. Outside the treatment field, it might reach up to 10%, depending on the number of particles hitting the different regions. It has been reported elsewhere [16, 17] that the calculated dose distribution is within measurement uncertainty outside the field.

Our study has some limitations. We decided to compare three patient groups in order to report data from clinically applied treatment plans; however, differences due to variability in patient’s anatomy between the groups cannot be excluded. Indeed, 2 patients were referred to PBI at the CTL instead of MRL because of obesity, which is not an absolute contraindication for treatment at the MRL but might be a challenge due to the coil dimensions. Nevertheless, the mean BMI was similar between the three groups. In addition, different planning modalities (S&S IMRT for PBI at the MRL, VMAT-IMRT for PBI at the CTL and dMLC-IMRT for WBI at the CTL) might also represent a bias in our analysis. We considered 33 patients, which is a limited number. Nevertheless, here it should be noted that most of the studies regarding RISM risk estimation after breast irradiation are plan comparison investigations performed in phantoms [46, 47] or for a very limited patient number between 10 and 20 [28, 45]. Non-inferiority of PBI compared to WBI has been proved with high-level of evidence for multicatheter brachytherapy [49] and external beam radiotherapy [6]. In our department, brachytherapy for PBI is not available. A comparison with second cancer risk after brachytherapy is therefore not presented, as we compared breast irradiation at the MRL and at the conventional linac, which represents the standard PBI technique in our department and is broadly available worldwide.

A strength of the present study is that the additional dose of the CBCT for PBI treatments at the CTL has been taken into account, where the component for the risk of RISM coming from IGRT modalities is usually not considered in the literature. This is particularly important for PBI, where, in order to guarantee precise treatment of a smaller target compared to the whole breast, a daily CBCT represents standard of care. Patients treated at the MRL are not exposed to this additional dose. We did not consider the dose due to the planning CT, since this is currently performed the same for patients treated at the MRL and CTL. Nevertheless, in the near future a MR-only based planning procedure, through the generation of a synthetic CT for planning, might be used, reducing even more the dose of ionizing radiation to which patients treated at the MRL are exposed.

Together with our previous publications [16, 17], the present paper provides evidence that the adjuvant PBI at the MRL is feasible and safe. Advantages of partial breast treatments at the MRL might be expected for highly hypofractionated PBI (e.g., according to Florence [38] or FAST forward [40] protocols) and particularly for neoadjuvant PBI [18]. To the best of our knowledge, no clinical data regarding highly hypofractionated or neoadjuvant PBI performed at the MRL have been published, but trials are open and recruiting [30, 32–35].

In conclusion, radiotherapy at the 1.5 T MRL is particularly attractive for breast cancer patients because of the better breast tissue visualization and daily plan adaption possibility. Our data suggest that this treatment can be performed without additional projected risk in terms of radiation-induced cancer.

## Supplementary Information

**Table 1 supplementary material**: Cone beam computed tomography characteristics

**Table 2 supplementary material**: Tumor location, clinical target volumes, planning target volumes (cc) and maximum mean dose to the contralateral breast for all patients

**Table 3 supplementary material**: Mean dose (Gy) for all organs for patients treated with PBI at the CTL without CBCT and considering a daily (15 in total) CBCTs.

**Table 4 supplementary material**: Mean dose (Gy) for all organs for patients treated with WBI at the CTL without CBCT and considering a total of 6 CBCTs.

**Fig. 1 supplementary material: A** dose distribution in a patient treated with PBI at the MRL. **B** dose distribution in a patient treated with PBI at the CTL. Structures depicted are: CTV, PTV, clips (*yellow* in **A** and *blue* in **B**), Seroma if present (in **B**), lungs and breasts ipsilateral and contralateral.

**Fig. 2 supplementary material**: Comparison of the planning target volumes (cc). Mean with standard deviation are shown. *P* = 0.6.

**Fig. 3 supplementary material**: Comparison of the maximum dose received by the contralateral breast. Mean with standard deviation are shown. *P*-values: PBI at the MRL vs PBI at the CTL *p* = 1.13; PBI at the MRL vs WBI at the CTL *p* = 0.015; PBI at the CTL vs WBI at the CTL (*p* = 0.026).
