# Supplementary material for: Estimation of secondary cancer projected risk after partial breast irradiation at the 1.5 T MR-linac
Source: Strahlenther Onkol. 2022 Apr 12;198(7):622–9. doi: 10.1007/s00066-022-01930-5 (PMC9217770; doi:10.1007/s00066-022-01930-5)
Supplement: Supplementary file 1 — Table 1 supplementary material: Cone beam computed tomography characteristics [file 66_2022_1930_MOESM1_ESM.pptx]

## Slide 1
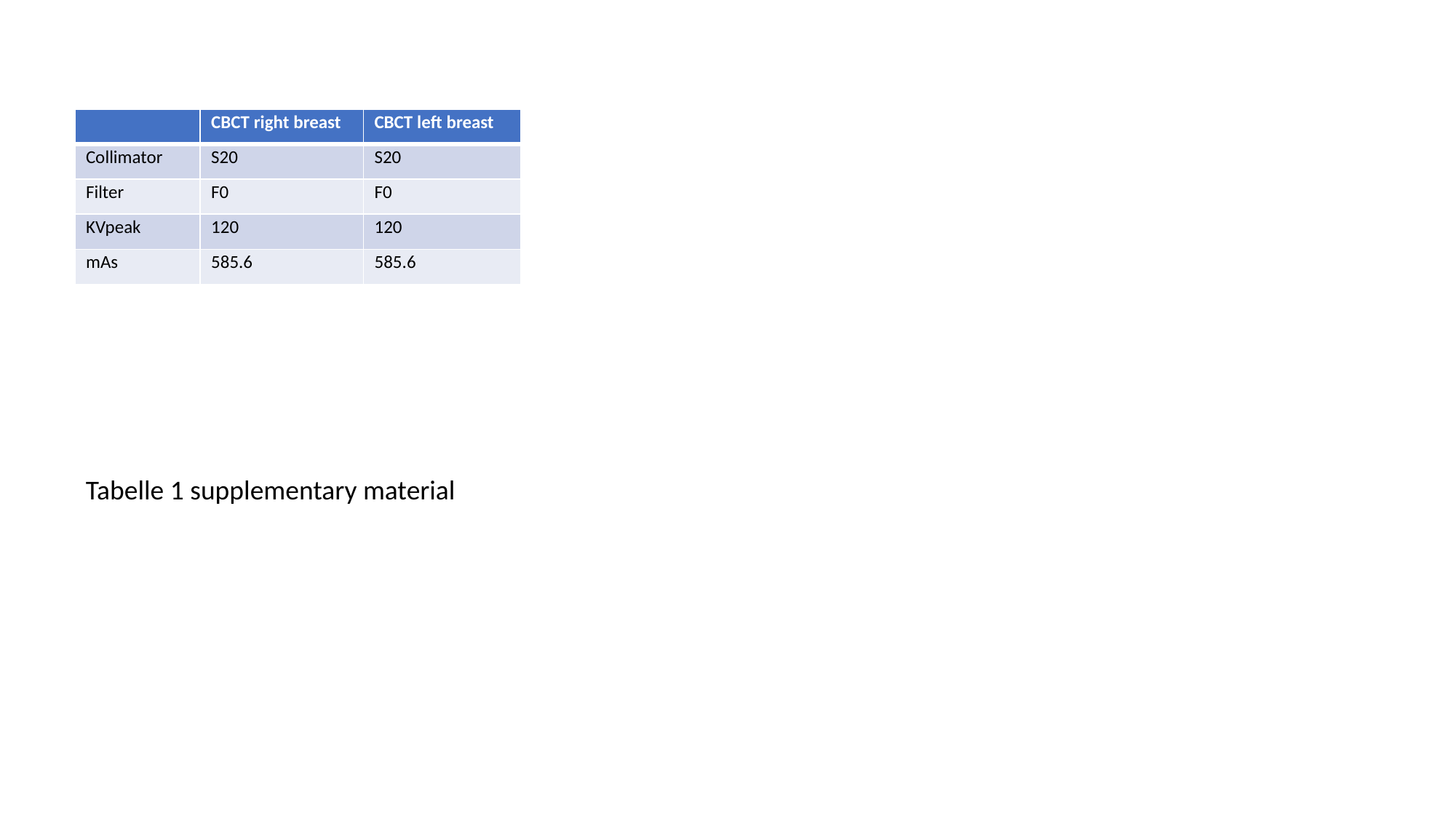

| | CBCT right breast | CBCT left breast |
| --- | --- | --- |
| Collimator | S20 | S20 |
| Filter | F0 | F0 |
| KVpeak | 120 | 120 |
| mAs | 585.6 | 585.6 |
Tabelle 1 supplementary material
