# Supplementary material for: Estimation of secondary cancer projected risk after partial breast irradiation at the 1.5 T MR-linac
Source: Strahlenther Onkol. 2022 Apr 12;198(7):622–9. doi: 10.1007/s00066-022-01930-5 (PMC9217770; doi:10.1007/s00066-022-01930-5)
Supplement: Supplementary file 2 — Table 2 supplementary material: Tumor location, clinical target volumes, planning target volumes (cc) and maximum mean dose to the contralateral breast for all patients [file 66_2022_1930_MOESM2_ESM.pptx]

## Slide 1
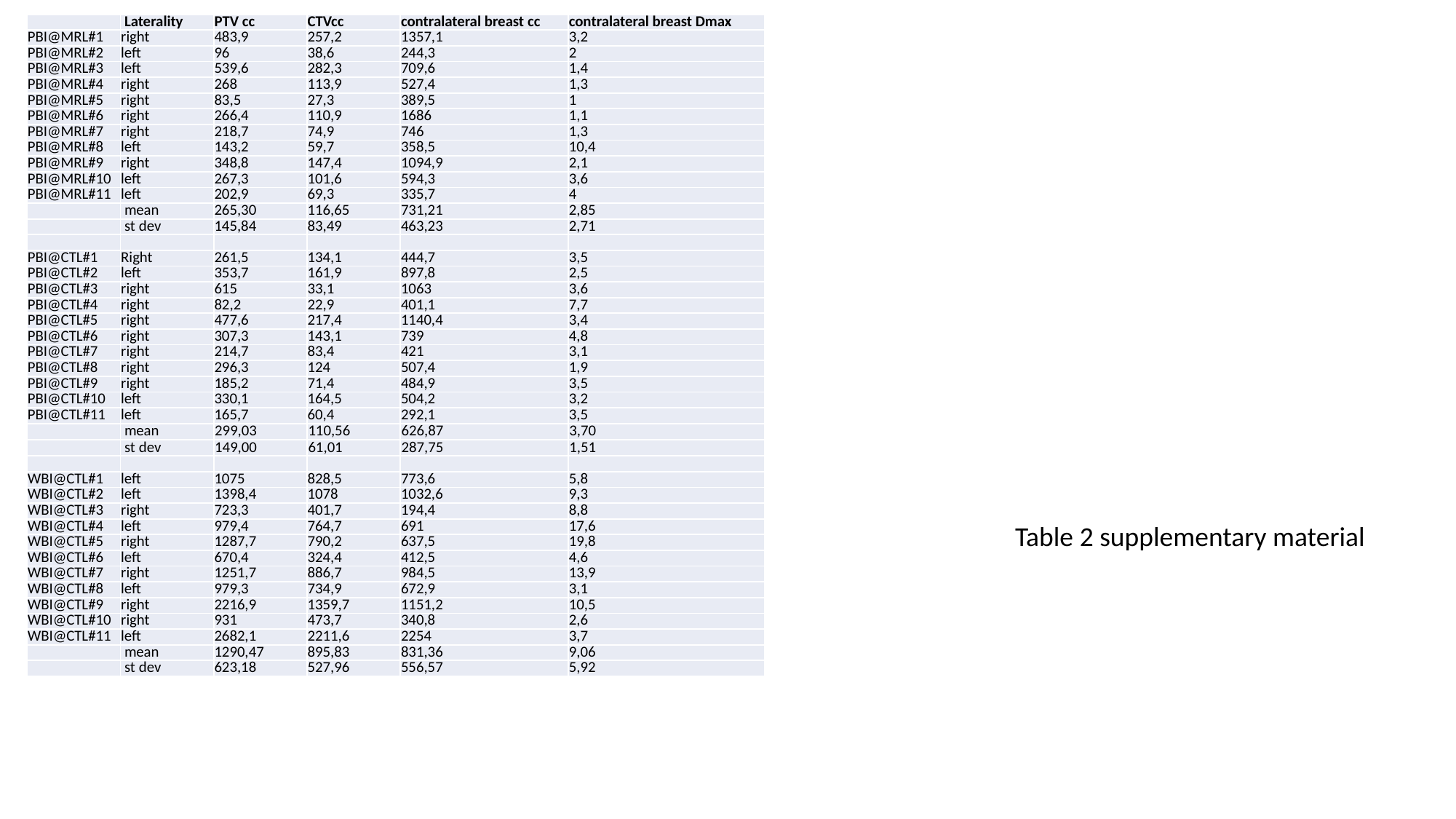

| | Laterality | PTV cc | CTVcc | contralateral breast cc | contralateral breast Dmax |
| --- | --- | --- | --- | --- | --- |
| PBI@MRL#1 | right | 483,9 | 257,2 | 1357,1 | 3,2 |
| PBI@MRL#2 | left | 96 | 38,6 | 244,3 | 2 |
| PBI@MRL#3 | left | 539,6 | 282,3 | 709,6 | 1,4 |
| PBI@MRL#4 | right | 268 | 113,9 | 527,4 | 1,3 |
| PBI@MRL#5 | right | 83,5 | 27,3 | 389,5 | 1 |
| PBI@MRL#6 | right | 266,4 | 110,9 | 1686 | 1,1 |
| PBI@MRL#7 | right | 218,7 | 74,9 | 746 | 1,3 |
| PBI@MRL#8 | left | 143,2 | 59,7 | 358,5 | 10,4 |
| PBI@MRL#9 | right | 348,8 | 147,4 | 1094,9 | 2,1 |
| PBI@MRL#10 | left | 267,3 | 101,6 | 594,3 | 3,6 |
| PBI@MRL#11 | left | 202,9 | 69,3 | 335,7 | 4 |
| | mean | 265,30 | 116,65 | 731,21 | 2,85 |
| | st dev | 145,84 | 83,49 | 463,23 | 2,71 |
| | | | | | |
| PBI@CTL#1 | Right | 261,5 | 134,1 | 444,7 | 3,5 |
| PBI@CTL#2 | left | 353,7 | 161,9 | 897,8 | 2,5 |
| PBI@CTL#3 | right | 615 | 33,1 | 1063 | 3,6 |
| PBI@CTL#4 | right | 82,2 | 22,9 | 401,1 | 7,7 |
| PBI@CTL#5 | right | 477,6 | 217,4 | 1140,4 | 3,4 |
| PBI@CTL#6 | right | 307,3 | 143,1 | 739 | 4,8 |
| PBI@CTL#7 | right | 214,7 | 83,4 | 421 | 3,1 |
| PBI@CTL#8 | right | 296,3 | 124 | 507,4 | 1,9 |
| PBI@CTL#9 | right | 185,2 | 71,4 | 484,9 | 3,5 |
| PBI@CTL#10 | left | 330,1 | 164,5 | 504,2 | 3,2 |
| PBI@CTL#11 | left | 165,7 | 60,4 | 292,1 | 3,5 |
| | mean | 299,03 | 110,56 | 626,87 | 3,70 |
| | st dev | 149,00 | 61,01 | 287,75 | 1,51 |
| | | | | | |
| WBI@CTL#1 | left | 1075 | 828,5 | 773,6 | 5,8 |
| WBI@CTL#2 | left | 1398,4 | 1078 | 1032,6 | 9,3 |
| WBI@CTL#3 | right | 723,3 | 401,7 | 194,4 | 8,8 |
| WBI@CTL#4 | left | 979,4 | 764,7 | 691 | 17,6 |
| WBI@CTL#5 | right | 1287,7 | 790,2 | 637,5 | 19,8 |
| WBI@CTL#6 | left | 670,4 | 324,4 | 412,5 | 4,6 |
| WBI@CTL#7 | right | 1251,7 | 886,7 | 984,5 | 13,9 |
| WBI@CTL#8 | left | 979,3 | 734,9 | 672,9 | 3,1 |
| WBI@CTL#9 | right | 2216,9 | 1359,7 | 1151,2 | 10,5 |
| WBI@CTL#10 | right | 931 | 473,7 | 340,8 | 2,6 |
| WBI@CTL#11 | left | 2682,1 | 2211,6 | 2254 | 3,7 |
| | mean | 1290,47 | 895,83 | 831,36 | 9,06 |
| | st dev | 623,18 | 527,96 | 556,57 | 5,92 |
Table 2 supplementary material
