# Supplementary material for: Estimation of secondary cancer projected risk after partial breast irradiation at the 1.5 T MR-linac
Source: Strahlenther Onkol. 2022 Apr 12;198(7):622–9. doi: 10.1007/s00066-022-01930-5 (PMC9217770; doi:10.1007/s00066-022-01930-5)
Supplement: Supplementary file 3 — Table 3 supplementary material: Mean dose (Gy) for all organs for patients treated with PBI at the CTL without CBCT and considering a daily (15 in total) CBCTs. [file 66_2022_1930_MOESM3_ESM.pptx]

## Slide 1
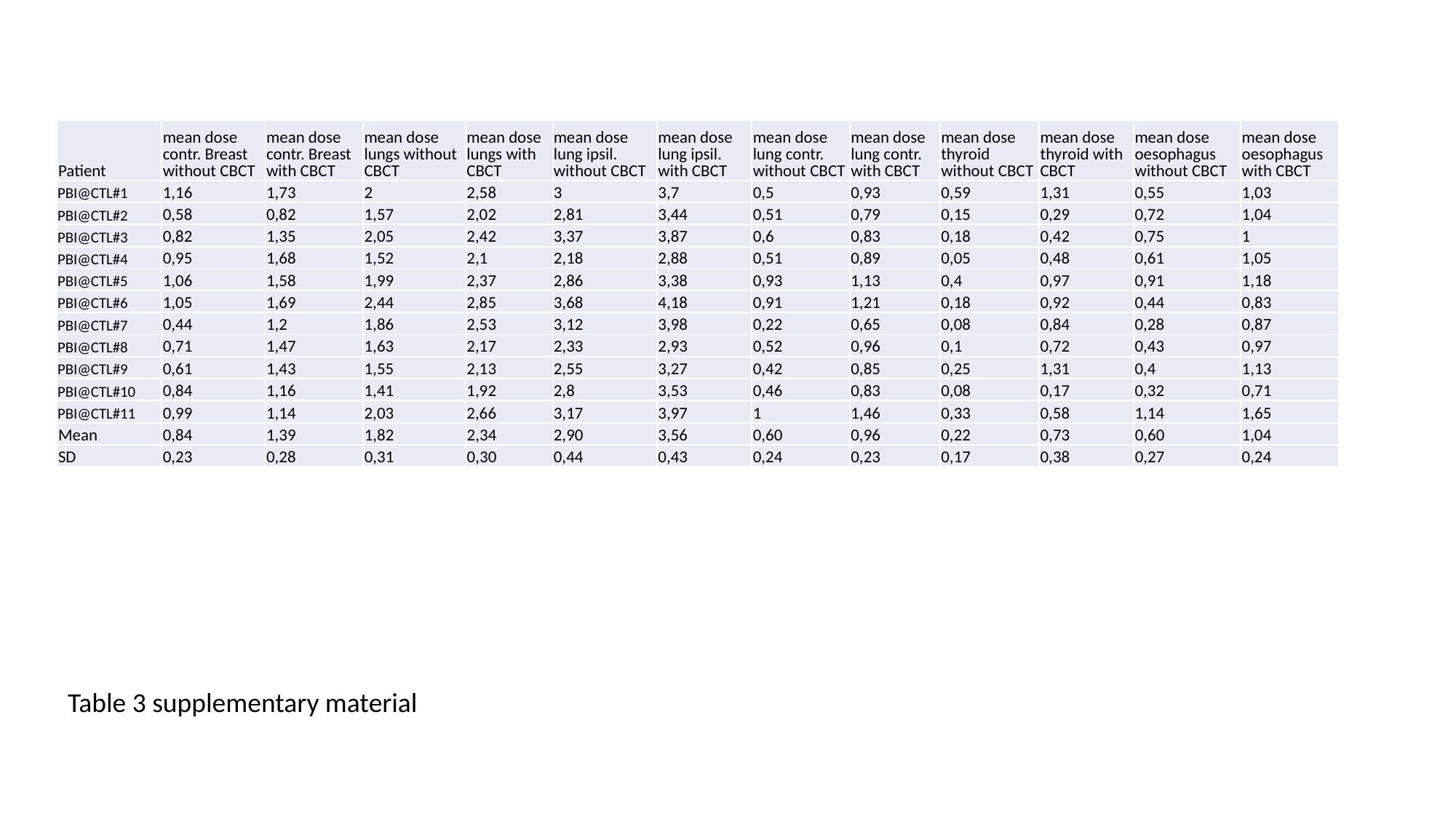

| Patient | mean dose contr. Breast without CBCT | mean dose contr. Breast with CBCT | mean dose lungs without CBCT | mean dose lungs with CBCT | mean dose lung ipsil. without CBCT | mean dose lung ipsil. with CBCT | mean dose lung contr. without CBCT | mean dose lung contr. with CBCT | mean dose thyroid without CBCT | mean dose thyroid with CBCT | mean dose oesophagus without CBCT | mean dose oesophagus with CBCT |
| --- | --- | --- | --- | --- | --- | --- | --- | --- | --- | --- | --- | --- |
| PBI@CTL#1 | 1,16 | 1,73 | 2 | 2,58 | 3 | 3,7 | 0,5 | 0,93 | 0,59 | 1,31 | 0,55 | 1,03 |
| PBI@CTL#2 | 0,58 | 0,82 | 1,57 | 2,02 | 2,81 | 3,44 | 0,51 | 0,79 | 0,15 | 0,29 | 0,72 | 1,04 |
| PBI@CTL#3 | 0,82 | 1,35 | 2,05 | 2,42 | 3,37 | 3,87 | 0,6 | 0,83 | 0,18 | 0,42 | 0,75 | 1 |
| PBI@CTL#4 | 0,95 | 1,68 | 1,52 | 2,1 | 2,18 | 2,88 | 0,51 | 0,89 | 0,05 | 0,48 | 0,61 | 1,05 |
| PBI@CTL#5 | 1,06 | 1,58 | 1,99 | 2,37 | 2,86 | 3,38 | 0,93 | 1,13 | 0,4 | 0,97 | 0,91 | 1,18 |
| PBI@CTL#6 | 1,05 | 1,69 | 2,44 | 2,85 | 3,68 | 4,18 | 0,91 | 1,21 | 0,18 | 0,92 | 0,44 | 0,83 |
| PBI@CTL#7 | 0,44 | 1,2 | 1,86 | 2,53 | 3,12 | 3,98 | 0,22 | 0,65 | 0,08 | 0,84 | 0,28 | 0,87 |
| PBI@CTL#8 | 0,71 | 1,47 | 1,63 | 2,17 | 2,33 | 2,93 | 0,52 | 0,96 | 0,1 | 0,72 | 0,43 | 0,97 |
| PBI@CTL#9 | 0,61 | 1,43 | 1,55 | 2,13 | 2,55 | 3,27 | 0,42 | 0,85 | 0,25 | 1,31 | 0,4 | 1,13 |
| PBI@CTL#10 | 0,84 | 1,16 | 1,41 | 1,92 | 2,8 | 3,53 | 0,46 | 0,83 | 0,08 | 0,17 | 0,32 | 0,71 |
| PBI@CTL#11 | 0,99 | 1,14 | 2,03 | 2,66 | 3,17 | 3,97 | 1 | 1,46 | 0,33 | 0,58 | 1,14 | 1,65 |
| Mean | 0,84 | 1,39 | 1,82 | 2,34 | 2,90 | 3,56 | 0,60 | 0,96 | 0,22 | 0,73 | 0,60 | 1,04 |
| SD | 0,23 | 0,28 | 0,31 | 0,30 | 0,44 | 0,43 | 0,24 | 0,23 | 0,17 | 0,38 | 0,27 | 0,24 |
Table 3 supplementary material
