# Supplementary material for: Estimation of secondary cancer projected risk after partial breast irradiation at the 1.5 T MR-linac
Source: Strahlenther Onkol. 2022 Apr 12;198(7):622–9. doi: 10.1007/s00066-022-01930-5 (PMC9217770; doi:10.1007/s00066-022-01930-5)
Supplement: Supplementary file 4 — Table 4 supplementary material: Mean dose (Gy) for all organs for patients treated with WBI at the CTL without CBCT and considering a total of 6 CBCTs. [file 66_2022_1930_MOESM4_ESM.pptx]

## Slide 1
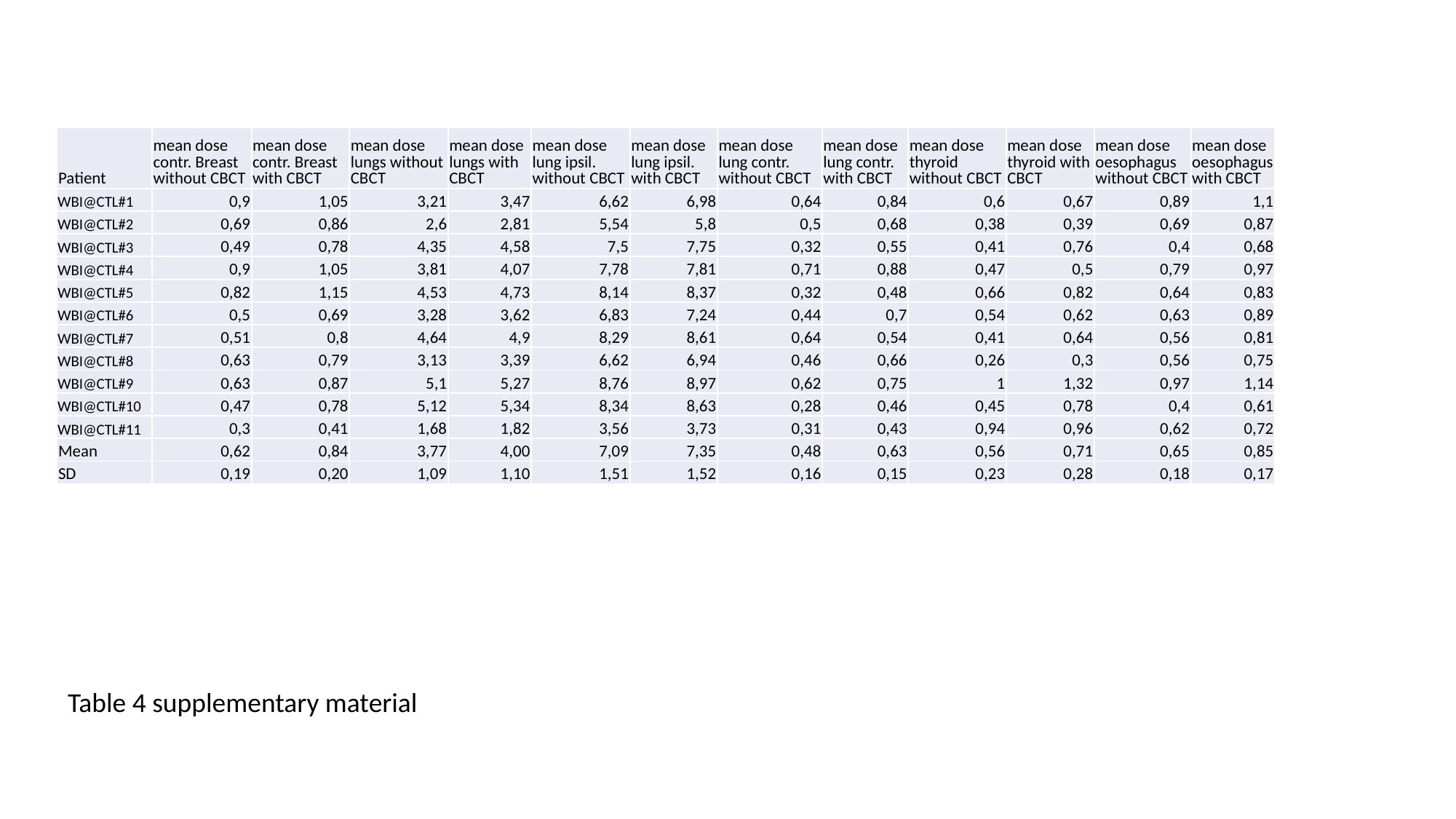

| Patient | mean dose contr. Breast without CBCT | mean dose contr. Breast with CBCT | mean dose lungs without CBCT | mean dose lungs with CBCT | mean dose lung ipsil. without CBCT | mean dose lung ipsil. with CBCT | mean dose lung contr. without CBCT | mean dose lung contr. with CBCT | mean dose thyroid without CBCT | mean dose thyroid with CBCT | mean dose oesophagus without CBCT | mean dose oesophagus with CBCT |
| --- | --- | --- | --- | --- | --- | --- | --- | --- | --- | --- | --- | --- |
| WBI@CTL#1 | 0,9 | 1,05 | 3,21 | 3,47 | 6,62 | 6,98 | 0,64 | 0,84 | 0,6 | 0,67 | 0,89 | 1,1 |
| WBI@CTL#2 | 0,69 | 0,86 | 2,6 | 2,81 | 5,54 | 5,8 | 0,5 | 0,68 | 0,38 | 0,39 | 0,69 | 0,87 |
| WBI@CTL#3 | 0,49 | 0,78 | 4,35 | 4,58 | 7,5 | 7,75 | 0,32 | 0,55 | 0,41 | 0,76 | 0,4 | 0,68 |
| WBI@CTL#4 | 0,9 | 1,05 | 3,81 | 4,07 | 7,78 | 7,81 | 0,71 | 0,88 | 0,47 | 0,5 | 0,79 | 0,97 |
| WBI@CTL#5 | 0,82 | 1,15 | 4,53 | 4,73 | 8,14 | 8,37 | 0,32 | 0,48 | 0,66 | 0,82 | 0,64 | 0,83 |
| WBI@CTL#6 | 0,5 | 0,69 | 3,28 | 3,62 | 6,83 | 7,24 | 0,44 | 0,7 | 0,54 | 0,62 | 0,63 | 0,89 |
| WBI@CTL#7 | 0,51 | 0,8 | 4,64 | 4,9 | 8,29 | 8,61 | 0,64 | 0,54 | 0,41 | 0,64 | 0,56 | 0,81 |
| WBI@CTL#8 | 0,63 | 0,79 | 3,13 | 3,39 | 6,62 | 6,94 | 0,46 | 0,66 | 0,26 | 0,3 | 0,56 | 0,75 |
| WBI@CTL#9 | 0,63 | 0,87 | 5,1 | 5,27 | 8,76 | 8,97 | 0,62 | 0,75 | 1 | 1,32 | 0,97 | 1,14 |
| WBI@CTL#10 | 0,47 | 0,78 | 5,12 | 5,34 | 8,34 | 8,63 | 0,28 | 0,46 | 0,45 | 0,78 | 0,4 | 0,61 |
| WBI@CTL#11 | 0,3 | 0,41 | 1,68 | 1,82 | 3,56 | 3,73 | 0,31 | 0,43 | 0,94 | 0,96 | 0,62 | 0,72 |
| Mean | 0,62 | 0,84 | 3,77 | 4,00 | 7,09 | 7,35 | 0,48 | 0,63 | 0,56 | 0,71 | 0,65 | 0,85 |
| SD | 0,19 | 0,20 | 1,09 | 1,10 | 1,51 | 1,52 | 0,16 | 0,15 | 0,23 | 0,28 | 0,18 | 0,17 |
Table 4 supplementary material
