# Supplementary figures and images for: Estimation of secondary cancer projected risk after partial breast irradiation at the 1.5 T MR-linac
Source: Strahlenther Onkol. 2022 Apr 12;198(7):622–9. doi: 10.1007/s00066-022-01930-5 (PMC9217770; doi:10.1007/s00066-022-01930-5)

## Slide 1
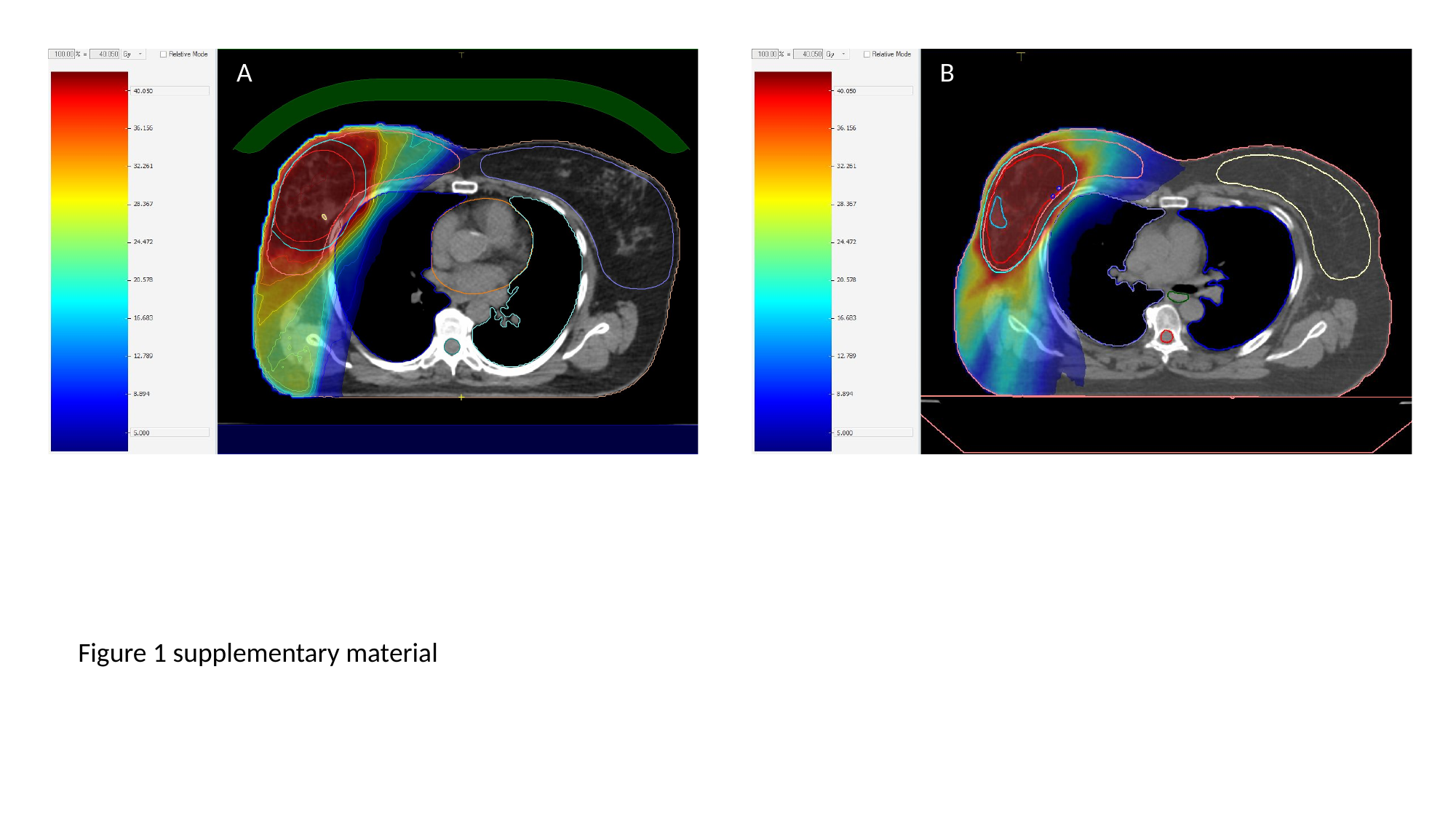

A
B
Figure 1 supplementary material

Supplement: Supplementary file 5 — Fig. 1 supplementary material: A dose distribution in a patient treated with PBI at the MRL. B dose distribution in a patient treated with PBI at the CTL. Structures depicted are: CTV, PTV, clips (yellow in A and blue in B), Seroma if present (in B), lungs and breasts ipsilateral and contralateral. [file 66_2022_1930_MOESM5_ESM.pptx]

## Slide 1
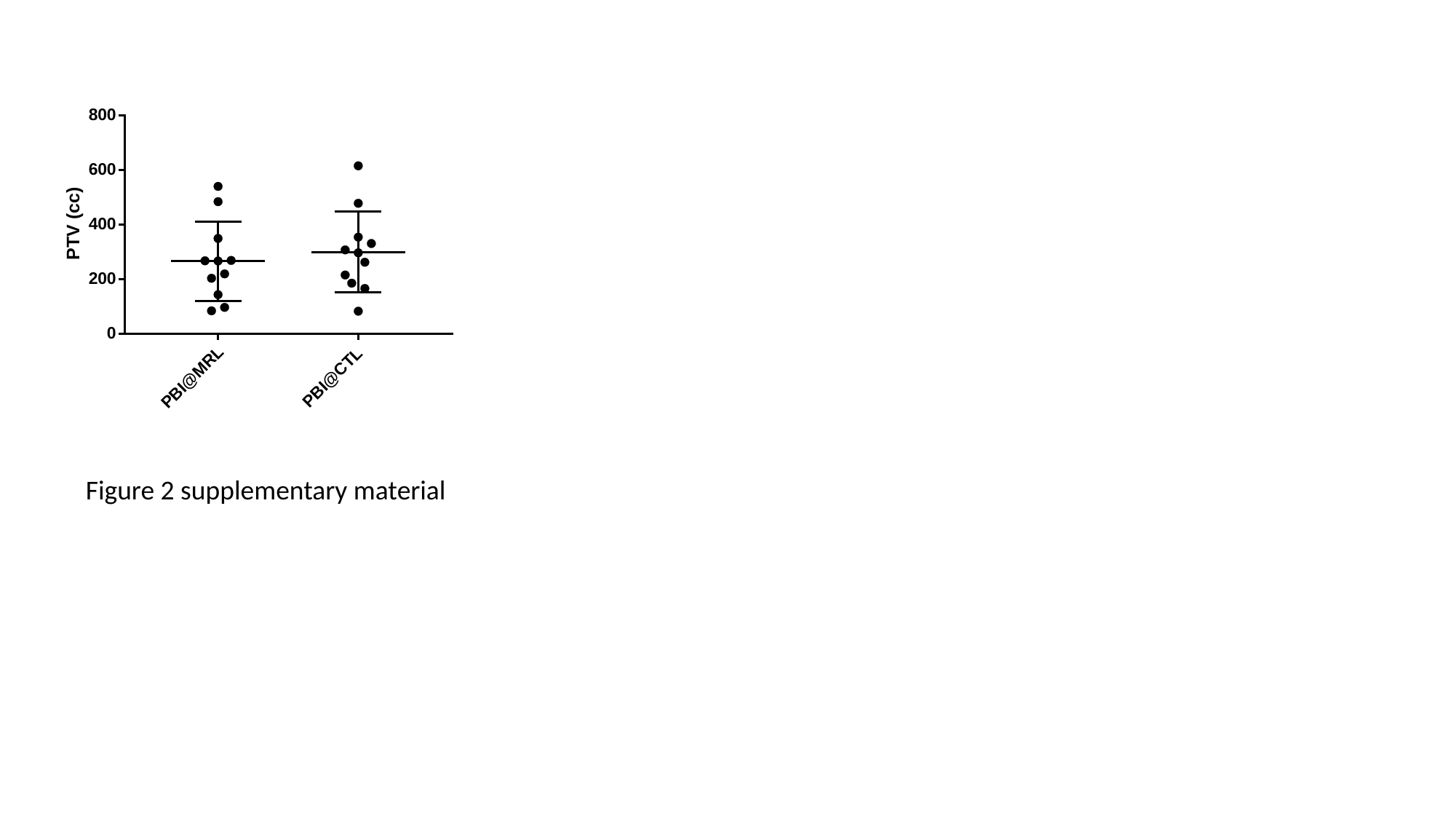

Figure 2 supplementary material

Supplement: Supplementary file 6 — Fig. 2 supplementary material: Comparison of the planning target volumes (cc). Mean with standard deviation are shown. P = 0.6. [file 66_2022_1930_MOESM6_ESM.pptx]

## Slide 1
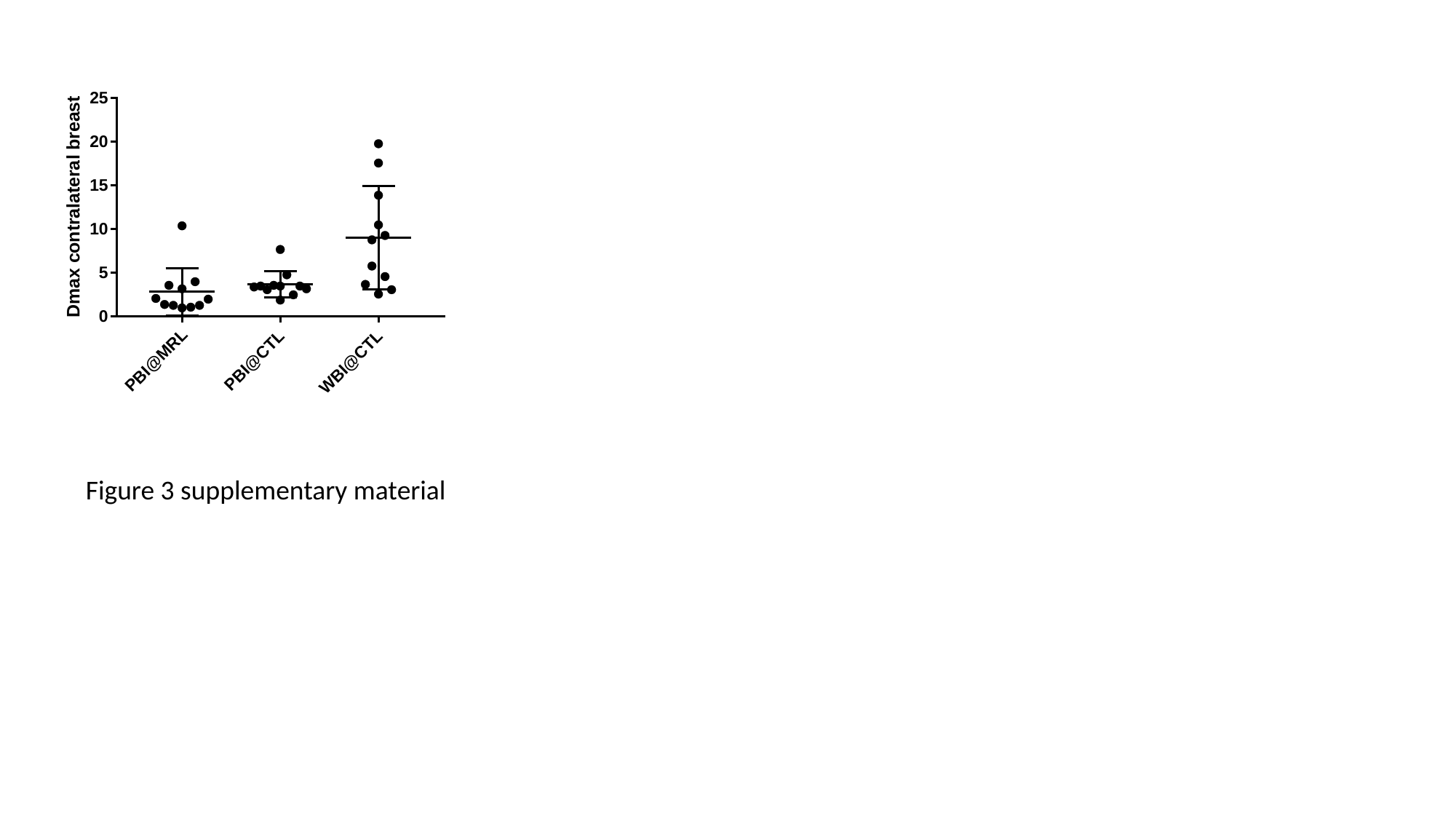

Figure 3 supplementary material

Supplement: Supplementary file 7 — Fig. 3 supplementary material: Comparison of the maximum dose received by the contralateral breast. Mean with standard deviation are shown. P-values: PBI at the MRL vs PBI at the CTL p = 1.13; PBI at the MRL vs WBI at the CTL p = 0.015; PBI at the CTL vs WBI at the CTL (p = 0.026). [file 66_2022_1930_MOESM7_ESM.pptx]
